# Passive Immunization With a Novel Monoclonal Anti-PrP Antibody TW1 in an Alzheimer’s Mouse Model With Tau Pathology

**DOI:** 10.3389/fnagi.2021.640677

**Published:** 2021-02-25

**Authors:** Allal Boutajangout, Wei Zhang, Justin Kim, Wed Ali Abdali, Frances Prelli, Thomas Wisniewski

**Affiliations:** ^1^Center for Cognitive Neurology, New York University Langone Health, New York, NY, United States; ^2^Department of Neurology, New York University Langone Health, New York, NY, United States; ^3^Department of Pathology, New York University Langone Health, New York, NY, United States; ^4^Department of Physiology and Neuroscience, New York University Langone Health, New York, NY, United States; ^5^Key Laboratory of Brain Functional Genomics (Ministry of Education) Shanghai, School of Life Sciences, East China Normal University, Shanghai, China; ^6^Department of Psychiatry, New York University Langone Health, New York, NY, United States

**Keywords:** Alzheimer’s disease, immunotherapy, prion, tau related pathology, passive immunization, transgenic mice

## Abstract

Neurofibrillary tangles (NFTs) are a major pathologic hallmark of Alzheimer’s disease (AD). Several studies have shown that amyloid β oligomers (Aβo) and tau oligomers mediate their toxicity, in part, *via* binding to cellular prion protein (PrP^C^) and that some anti-PrP antibodies can block this interaction. We have generated a novel monoclonal anti-PrP antibody (TW1) and assessed the efficacy of passive immunization with it in a mouse model of AD with extensive tau pathology: hTau/PS1 transgenic (Tg) mice. These mice were injected intraperitoneally once a week with TW1 starting at 5 months of age. Behavior was assessed at 8 months of age and brain tissue was subsequently harvested for analysis of treatment efficacy at 9 months. Mice treated with TW1 did not show any significant difference in sensorimotor testing including traverse beam, rotarod, and locomotor activity compared to controls. Significant cognitive benefits were observed with the novel object recognition test (ORT) in the immunized mice (two-tailed, *t*-test *p* = 0.0019). Immunized mice also showed cognitive benefits on the closed field symmetrical maze (day 1 two-tailed *t*-test *p* = 0.0001; day 2 two-tailed *t*-test *p* = 0.0015; day 3 two-tailed *t*-test *p* = 0.0002). Reduction of tau pathology was observed with PHF-1 immunohistochemistry in the piriform cortex by 60% (two-tailed *t*-test *p* = 0.01) and in the dentate gyrus by 50% (two-tailed *t*-test *p* = 0.02) in animals treated with TW1 compared to controls. There were no significant differences in astrogliosis or microgliosis observed between treated and control mice. As assessed by Western blots using PHF-1, the TW1 therapy reduced phosphorylated tau pathology (two-tailed *t*-test *p* = 0.03) and improved the ratio of pathological soluble tau to tubulin (PHF1/tubulin; two-tailed *t*-test *p* = 0.0006). Reduction of tau pathology also was observed using the CP13 antibody (two-tailed *t*-test *p* = 0.0007). These results indicate that passive immunization with the TW1 antibody can significantly decrease tau pathology as assessed by immunohistochemical and biochemical methods, resulting in improved cognitive function in a tau transgenic mouse model of AD.

## Introduction

Alzheimer’s disease (AD) is a devastating age-related neurodegenerative disorder characterized by forming toxic oligomers that eventually deposit as insoluble amyloid plaques and neurofibrillary tangles (NFTs). There are no disease-modifying therapeutic interventions for AD (Long and Holtzman, 2019; Wisniewski and Drummond, 2019). However, immunotherapy is emerging as a promising therapy to treat AD (Kwon et al., 2020). Numerous studies of passive immunization with anti-amyloid (Aβ) antibodies have demonstrated effective clearance of amyloid Aβ together with cognitive improvements in transgenic animal models (Herline et al., 2018a; Kwon et al., 2020). However, active and passive immunization targeting Aβ has failed in all clinical trials thus far, with the possible recent exception of aducanumab (Loureiro et al., 2020; Plotkin and Cashman, 2020), an antibody that is more specific to insoluble fibrillary and oligomeric forms of Aβ (Arndt et al., 2018; Tolar et al., 2020). There is a growing realization that the most toxic species of Aβ are oligomeric (Viola and Klein, 2015); similarly, it is also thought that oligomeric species of other proteins (i.e., tau, α-synuclein, TDP-43, etc.) that accumulate in association with neurodegenerative diseases are the most neurotoxic (Kwon et al., 2020). Hence it has been proposed that the inhibition of oligomer mediated toxicity by immunotherapy is the most likely approach to be clinically successful, for AD and other neurodegenerative disorders (Wisniewski and Goñi, 2015; Herline et al., 2018a). Tau targeted immunotherapies are currently also being developed; however, less effort has been directed on interventions targeting tau pathology compared to Aβ (Li and Götz, 2017; Götz and Götz, 2019; Kwon et al., 2020; Vaz and Silvestre, 2020). In the present study, we aimed to test a novel immunotherapeutic approach’s ability to ameliorate tau-related pathology by blocking oligomer mediated toxicity, using an AD transgenic (Tg) model, which we developed (Boutajangout et al., 2008, 2009, 2010). It has been established that tau-related pathology and the burden of NFTs correlates much better with cognitive dysfunction, compared to the amyloid beta burden (Nelson et al., 2012; Malpas et al., 2020); hence this AD pathology may represent a more important target to achieve successful clinical translatability. Previously, we have published for the first time that prophylactic active and passive immunization clear tau aggregates from different areas of the brain and prevent cognitive decline in two different models of AD (Asuni et al., 2007; Boutajangout et al., 2010; Wisniewski and Boutajangout, 2010; Boutajangout et al., 2011). In clinical trials, several ongoing passive immunotherapy targeting tau reached phase clinical trials (Novak et al., 2018a,b; Boxer et al., 2019; Kwon et al., 2020; Vaz and Silvestre, 2020).

Several studies showed that amyloid β oligomers (Aβo) mediate their toxicity, in part, *via* binding to cellular prion protein (PrP^C^) on the surface of neurons (Jarosz-Griffiths et al., 2016; Smith et al., 2019), with more recent data suggesting that other oligomeric species including those of tau and α-synuclein also mediate toxicity *via* interaction with PrP^C^ (Corbett et al., 2020). Previously, it has been also reported that short-term treatment with an anti-PrP antibody, 6D11, in AD model APP/PS1 mice can dramatically reverse behavioral deficits without affecting the amyloid burden by blocking the Aβo/PrP^C^ interaction (Chung et al., 2010). A prior publication demonstrated that the 6D11 antibody was highly effective at treating prion infection in tissue culture and *in vivo* by blocking the interaction between PrP^C^ and PrP^Sc^ (Sadowski et al., 2009). It has also been reported that anti-PrP^C^ mAb 6D11 blocks the Aβo binding site on PrP^C^ preventing the impairment in long-term potentiation (LTP) caused by Aβo derived from AD brain extracts (Barry et al., 2011; Freir et al., 2011), suggesting that 6D11 and possibly antibodies with a similar epitope have therapeutic potential in multiple neurodegenerative diseases. In the present study, we have generated a novel monoclonal antibody anti-PrP antibody (TW1), which has an epitope on PrP that is similar to that of 6D11. Furthermore, in this study, we investigated the potential effect of passive immunization of TW1 in a mouse model of AD with tau pathology, by targeting the tau oligomer to PrP^C^ interaction.

## Materials and Methods

### Transgenic Mouse Model

We used a transgenic mouse model for AD that expresses mutated PS1 (M146L) and all human tau isoforms on a murine tau knockout background, which we developed (Boutajangout et al., 2009, 2010). This double transgenic mice model was developed by crossbreeding a human tau (Htau) model with mice that have an AD linked presenilin 1 (PS1) mutation (M146L). Htau mice were generated by selective mating of two previously generated mouse lines, 8c and tau KO mice. The 8c mice express a tau transgene, derived from a human PAC driven by the tau promoter, and produce all human tau isoforms (Andorfer et al., 2005). The promotor used in the PS1 mice is PDGF-β2 (Duff et al., 1996). The tau-related pathology in this model starts at 3 months and behavioral deficits are observed from 6 months of age (Boutajangout and Wisniewski, 2014). This model does not have amyloid-beta pathology. Both males and females were included in approximately equal ratios for all experiments (*n* = 10 per group). Animals were maintained on a 12 h light/dark schedule (lights on at 7 AM) in a specific pathogen-free facility. All procedures for animal experiments were approved by the Institutional Animal Care and Use Committee (IACUC) of New York University Langone Health.

### Production, Purification and Complete Sequence for a Monoclonal Antibody TW1

TW1 is a novel monoclonal antibody (mAb) raised against a non-denatured PK-resistant fragment of PrP^Sc^ that was purified from brains of CD-1 mice infected with the 139A mouse-adapted scrapie agent according to previously published protocols (Kascsak et al., 1986; Carp et al., 2000). Briefly, wild-type CD-1 mice, 2 months of age, were immunized subcutaneously with the purified PrP^Sc^ in Freund’s adjuvant as previously published (Sigurdsson et al., 2002). Hybridomas reactive to PrP^Sc^ were generated as previously described (Spinner et al., 2007; Goñi et al., 2017). TW1 was derived from these hybridomas using methods as previously described (Goñi et al., 2017; Herline et al., 2018b). In brief, the TW1 mAb was purified from concentrated tissue-culture supernatant produced in Integra CL bioreactor flasks (Integra Biosciences, Chur, Switzerland). On day 8, the culture supernatant was collected, centrifuged, and filtered. This was followed by loading on to MabSelect Columns (GE, cat. No. 17-5199-03). The loading proceeded at a flow rate of 10.0 ml/min, followed by appropriate washing and elution. The pooled fractions of the purified mAb were dialyzed to phosphate-buffered saline (PBS) pH 7.2. The purity and integrity of the TW1 mAb were analyzed by SDS-PAGE (Herline et al., 2018b). TW1 reactivity to both PrP^Sc^ and recombinant PrP^C^ was tested on Western blots using methods as previously described (Goñi et al., 2017). Also, the TW1 hybridoma cells were lysed and total RNA was isolated by using Trizol reagent, thereafter, mRNA was purified over a poly (dt) affinity column.

### Production of cDNA and Purification of DNA

The TW1 cDNA was produced from mRNA by reverse transcriptase and amplified by polymerase chain reaction (PCR; Biolabs Kit). cDNA fragments were amplified by using designed primers obtained from the Mouse IgG Library Primer set (Progen Biotechnik, Heidelberg, Germany) to amplify VH scFv: Forward primer (5′-TGAGGAGACGGTGACC GTGGTCCCTTGGCCCCAG-3′) and primer reverse (5′-AGGTSMARCTG CAGSAG TCWGG-3′), and primers for the amplification of VL scFv: Forward primer (5′-CCGTTTGAT CTCGAGCTTGGTGCC-3′) and reverse primer(5′- GACATCGAGCTCACTCAGTCTCCA-3′). The following PCR cycling conditions are: Initial denaturation, 95°C for 1 min; 30 cycles (94°C for 30 s, 55°C for 30 s, 68°C for 1 min) and final extension 68°C for 5 min.

PCR product was loaded on the agarose gel. Thereafter, DNA was extracted from the gel and purified by using a Qiagen kit, the elution product was quantified and sequenced.

### Sequencing of Variable Region of TW1 Antibody Heavy and Light Chain

**Figure 1:**
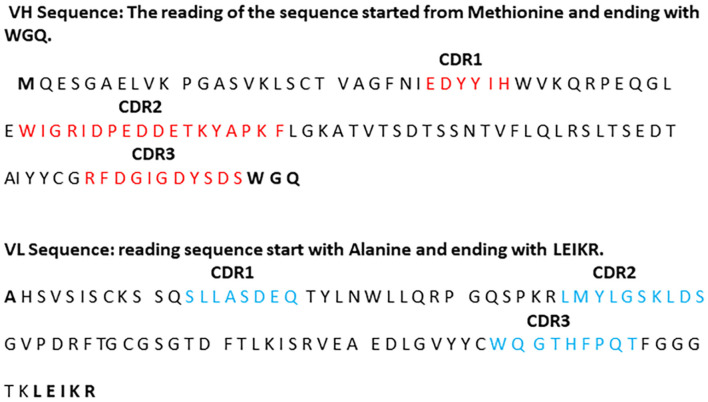
The sequence of TW1 antibody and the identification of the variable regions CDR1, CDR2, and CDR3 of the heavy chain and CDR1, CDR2, and CDR3 of the light chain.

Purified PCR products were quantified and sent to the Genewiz Company for DNA sequencing. The amino acid sequence of the variable region of TW1 heavy chain and light chain and their complementarity-determining regions (CDRs) were identified using resources available at the National Center for Biotechnology Information websites and determining the alignments of cDNA and amino acid sequences (Laune et al., 1997; Ofran et al., 2008; Figure 1). The variable region of the heavy chain (VH) contained 108 amino acids started from Methionine and ending with WGQ. The variable region of a light chain (VL) contained 98 amino acids started from Alanine and ending with LEIKR.

### Dot Blot for Epitope Mapping of TW1

PrP sequence peptides were synthesized on an ABI 430A peptide synthesizer (AME Bioscience, Chicago, IL, USA) at the Keck peptide synthesis facility at Yale University, New Haven, CT, USA. Mass spectroscopy of the lyophilized end-product was used to verify the expected molecular weight. For dot-blotting analysis, PrP peptides, 1 μg/100 μl/dot was spotted on nitrocellulose membrane. The membrane was blocked with 5% nonfat milk in TBST (10 mM Tris; 150 mM NaCl; 0.1% Tween 20, pH 7.5) for 1 h at room temperature (RT) and then washed with TBST. The membrane was then incubated with Mab TW1 diluted 1:100 for 1 h at RT. Following extensive washing, in TBST the membrane was incubated for 1 h with a horseradish peroxidase-conjugated sheep anti-mouse IgG antibody (Amersham) and then developed using an enhanced chemiluminescent substrate (SuperSignal; Pierce).

### Treatment With TW1 Antibody

hTau/PS1 transgenic AD model mice were injected intraperitoneally 200 μg once a week with our monoclonal anti-PrP antibody TW1 from the age of 5 months. Control mice received intraperitoneal injections of sterile saline alone (100 μl). Their behavior was assessed at 8 months of age and brain tissue was subsequently harvested for analysis of treatment efficacy at 9 months.

### Behavioral Tasks

#### Traverse Beam

This task tested balance, general motor coordination, and overall motor intentionality. It determined the ability to traverse a graded narrow wooden beam to reach a goal box and was done as previously described (Boutajangout et al., 2012, 2017, 2019). In brief, the mice were placed on a 1.1 cm wide beam 50.8 cm long that was suspended by two identical columns 30 cm above a padded surface. A shaded goal box was attached at each end of the beam. Mice were placed on the beam to habituate and were monitored for a maximum of 60 s. The number of foot slips before falling or reaching the goal box was recorded for each of four successive trials. Errors were defined as foot slips and recorded numerically. To prevent injury from falling, a soft foam cushion was kept underneath the beam. Animals that fall off were placed back in their position before the fall.

#### Rotarod

Each animal was placed on the rod (diameter 3.6 cm) apparatus to assess differences in motor coordination and balance by measuring fore- and hind-limb motor coordination and balance (Rotarod 7650 accelerating model; Ugo Basile, Biological Research Apparatus, Varese, Italy), as previously described (Boutajangout et al., 2012, 2019). This procedure was designed to assess motor behavior without a practice confound. The animals were habituated to the apparatus by receiving training sessions of two trials, sufficient to reach a baseline level of performance. Then the mice were tested three more times, with increasing speed. During habituation, the rotor rod was set at 1.0 rpm and gradually raised every 30 s. The rod was wiped clean with 30% ethanol solution after each session. A soft foam cushion was placed beneath the apparatus to prevent potential injury from falling. Each animal was tested for three sessions, with a break of 15 min between sessions. Measures were made of the latency between being placed on the rod and a fall or inversion (while clinging upside down on the rod).

### Locomotor Activity

A Hamelton-Kinder photobeam system was used to measure the activity of the animals, as previously described (Boutajangout et al., 2012, 2017, 2019). A video camera recorded the horizontal movements on the circular open field chamber (75 × 75 cm). Each animal was tested for 15 min, Results are reported based on distance traveled (cm), mean resting time, and velocity (average and maximum) of the animals.

### Cognitive Tests

#### Spontaneous Object Recognition Test

The spontaneous ORT that was used to measure changes in short term memory, and was conducted in a square-shaped open field box (48 cm square, with 18 cm high walls constructed from black Plexiglas), raised 50 cm from the floor, as previously described (Boutajangout et al., 2012, 2017, 2019). The light intensity was set to 30 lux. On the day before the tests, mice were individually habituated in a session in which they were allowed to explore the empty box for 15 min. During training sessions, two novel objects were placed at diagonal corners in the open field and the animal was allowed to explore for 15 min. The time spent exploring each object was recorded by a tracking system (San Diego Instruments, San Diego, CA, USA), and at the end of the training phase, the mouse was removed from the box for the duration of the retention delay (3 h). During retention tests, the animals were placed back into the same box, in which one of the previous familiar objects used during training was replaced by a novel object, and allowed to explore freely for 6 min. A different object pair was used for each trial for a given animal, and the order of exposure to object pairs as well as the designated sample and novel objects for each pair were counterbalanced within and across groups. The time spent exploring the novel and familiar objects were recorded for 6 min. The percentage short-term memory score is the time spent exploring any one of the two objects (training session) compared with the novel one (retention session).

#### Closed Field Symmetrical Maze

This apparatus is a rectangular field 30 cm square with 9 cm high walls divided into 36 9.5 cm squares and covered by a clear Plexiglas top. End boxes, each 11 × 16 × 9 cm, are situated at diagonal corners of the field. The symmetrical maze is a modification of the Hebb–Williams and Rabinovitch–Rosvold types of tests, as we have described previously (Asuni et al., 2006; Boutajangout et al., 2012, 2017, 2019). Briefly, the main difference is that each end compartment functions as both a start box and a goal box, and the mice run in opposite directions on alternate trials, thereby eliminating intertrial handling. The barriers are placed in the field in symmetrical patterns so that mice face the same turns going in either direction within a given problem. Before testing, the mice were adapted to a water-restriction schedule (2 h daily access to water). Each mouse (treated htau/PS1 and non-treated) was given two adaptation sessions before the beginning of testing. In the first session, all animals were given saccharine-flavored water in the goal box for 10 min. In session 2, they were placed in the start chamber and permitted to explore the field and enter the goal box where water reward (0.05 ml) was available. When the mice were running reliably from the start chamber to the goal box, they were given three practice sessions on simple problems where one or two barriers were placed in different positions in the field to obstruct direct access to the goal box. Formal testing consisted of the presentation of three problems graded for difficulty, based on our data and published norms for mice. One problem was presented per day, and the mice were given five trials on each problem with an intertrial interval of 2 min. The performance was scored manually by the same observer in terms of errors (i.e., entries and reentries into designated error zones) and time to complete each trial.

### Histology

The right hemisphere was immersion-fixed overnight in periodate-lysine-paraformaldehyde. Following fixation, the brains were transferred to a phosphate buffer solution containing 20% glycerol and 2% dimethylsulfoxide (DMSO) and stored at 4°C. Serial coronal brain sections (40 μm) were cut, placed in ethylene glycol cryoprotectant, and stored at −20°C. Serial coronal sections (40 μm) were cut and saved in ethylene glycol cryoprotectant at −20°C for further histological staining using a monoclonal antibody PHF-1 that recognizes epitopes Ser 396 and Ser 404, and a monoclonal antibody CP13 that recognizes phospho-epitope Ser 202 (PHF-1 and CP13 generously provided by Peter Davies). Inflammation was tested by anti-GFAP (1:1,000; Vector Laboratories Inc., CA, USA) detection of astrocytes and microglia activation was detected by Iba-1 antibody (1:1,000; Wako Chemical Industries). The brain sections from treated and controls mice were stained with PHF-1 and CP13. Semi-quantitative analysis was performed using a scale rating from 0 to 3, in increments of 0.5, depending on the degree of tau pathology in the motor cortex, dentate gyrus, and piriform cortex, by an observer blinded to the treatment status of the mice, as we have previously published (Scholtzova et al., 2014; Rubenstein et al., 2017; Scholtzova et al., 2019).

The assessment of sections stained with GFAP, and Iba-1 antibodies were based on a semi-quantitative analysis, using methods we have previously published (Boutajangout et al., 2012; Scholtzova et al., 2014; Boutajangout et al., 2017, 2019). Before analysis, brains were checked by microscope and given a rating from 0 to 4, in increments of 0.5, depending on the degree of pathology and/or the activation stage of the glial cells. Approximately five cortical sections and six hippocampal sections were analyzed per animal. The rating was based on the number of reactive neuronal bodies and processes. Astrogliosis and microgliosis were analyzed at 10× magnifications, respectively in the cortex, dentate gyrus, and piriform cortex. Representative images were taken at 20× magnification. The rating for astrogliosis was based on the extent of GFAP immunoreactivity (number of GFAP immunoreactive cells and complexity of astrocytic branching) as previously published. The assessment of microgliosis was based on the extent of immunoreactivity with Iba-1 antibody with a rating from 0 (few resting microglia) to 4 (numerous ramified/phagocytic microglia) in increments of 0.5. The rating was performed by an observer blinded to the treatment status of the mice, as previously described (Scholtzova et al., 2014; Boutajangout et al., 2017, 2019).

### Biochemistry

The left hemisphere from treated hTau/PS1 mice and controls was prepared as described (Boutajangout et al., 2011, 2017, 2019). Briefly, the homogenate was centrifuged (20,000× *g*) for 30 min at 4°C to separate a soluble cytosolic fraction (supernatant) and an insoluble fraction (pellet). The pellets were suspended in the same volume of the buffer without protease and kinase inhibitors and centrifuged at 50,000× *g* and the supernatant was analyzed as the insoluble fraction. The soluble and insoluble fractions were heated at 100°C for 5 min and equal protein aliquots were electrophoresed on 12.5% Tris/Tricine SDS polyacrylamide gel. Proteins were transferred for Western blot analysis on to nitrocellulose membranes and were incubated with anti-tubulin, phospho-tau PHF-1, and CP13 antibodies, and then the blots were washed and incubated at RT for 1 h with peroxidase-conjugated, anti-mouse (GE Healthcare UK Limited), or anti-rabbit IgG (GE Healthcare UK Limited). Subsequently, ECL (Pierce) detected the bound antibodies. Densitometry analysis of immunoblots was performed by the Alpha view protein simple program and the levels of pathological tau were normalized relative to the amounts of tubulin (Sigma–Aldrich).

### Statistical Analysis

All data were analyzed with GraphPad Prism 9. The behavioral testing using the traverse beam test and the rotarod test were analyzed by unpaired two-tailed *t*-test. Locomotor activity (distance traveled, maximum velocity, average speed, and resting time), tau aggregates on western blots, and immunoreactivity on brain sections within the dentate gyrus, motor cortex, and piriform cortex were analyzed with unpaired two-tailed or one-tailed *t*-test.

## Results

### Sequence of TW1 Antibody

The purified DNA was sequenced (Genewiz). The amino acid sequences of the heavy and the light chains variable region and CDRs of the anti-PrP antibody were obtained. Three variable regions were identified in heavy chain H-CDR1, HCDR2, and H-CDR3; three others were identified in the light chain L-CDR1, L-CDR2, and L-CDR3. The sequence of each variable region is shown in Figure 1.

### Characterization of TW1

**Figure 2:**
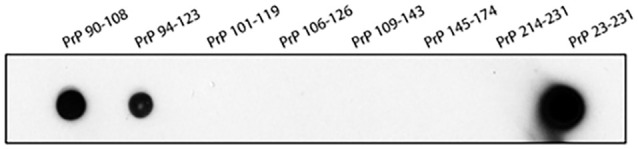
Epitope mapping of TW1. The epitope mapping was performed with dot blot that shows the immunoreactivity of TW1 antibody with human PrP sequences: PrP 90-108; PrP94-123 and PrP23-231. Other PrP sequences were not recognized by the TW1 antibody.

The epitope mapping was performed by dot blot that shows the immunoreactivity of TW1 antibody with mouse PrP sequences: PrP 90-108; PrP 94-123 and PrP 23-231. Other PrP sequences were not recognized by the TW1 antibody (Figure 2). The epitope recognized by TW1 is PrP 94-108. TW1 immunolabeled both recombinant PrP and PrP^Sc^ on Western blot (data not shown).

### Behavioral Tasks

**Figure 3:**
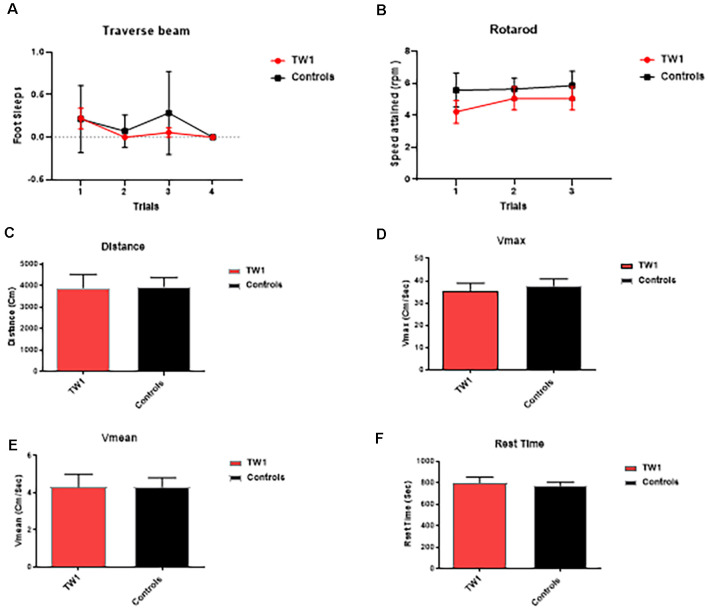
Tests of motor function. **(A)** Traverse beam: no difference was detected between controls and immunized animals in the number of foot slips in the Traverse Beam Test. **(B)** Rotarod: no significant difference was seen in speed attained (rpm) between controls and immunized animals. Locomotor activity: no differences were observed between groups in their **(C)** distance traveled, **(D)** maximum velocity, **(E)** average speed, or **(F)** resting time.

**Figure 4:**
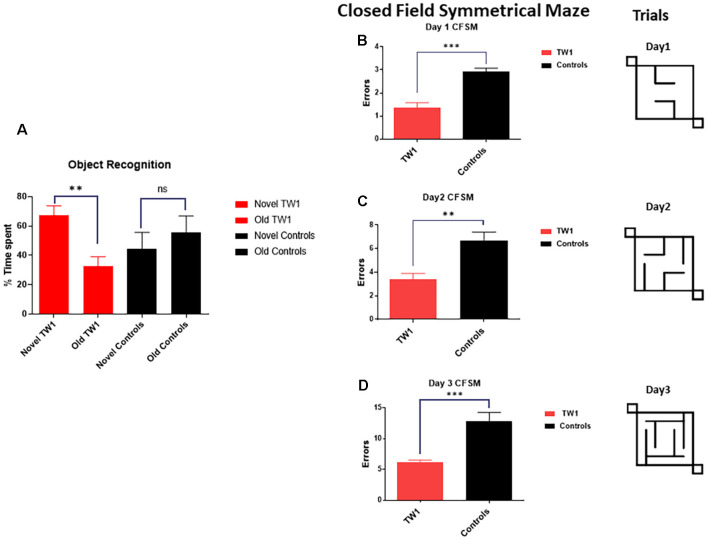
Passive immunotherapy with the TW1 antibody improves cognitive tasks in the hTau/PS1 Alzheimer’s disease (AD) mice model. Panel **(A)** shows working memory improvement using a short-term memory test (Novel Object Recognition Test). The bars depict the percentage of the amount of time spent with the novel or old object. Mice treated with TW1 spent more time with the novel object compared to the old object (two-tailed *t*-test, **p = 0.0019). Vehicle treated mice showed no significant difference in spent with the novel object. Panels **(B–D)** shows results on the Closed field Symmetrical Maze comparing TW1 treated mice vs. vehicle-treated mice: **(B)** Day 1 two-tailed *t*-test ***p < 0.0001; **(C)** Day 2 two-tailed *t*-test ** *p* = 0.0015; **(D)** Day 3 two-tailed *t*-test ***p = 0.0002). ns: not significant.

The behavioral tasks performed by controls and immunized animals did not show a significant difference in the number of foot slips in the traverse Beam or in the rotarod test (Figures 3A,B). The same results were observed with measures of locomotor activity: (Figure 3C) distance traveled; (Figure 3D) maximum velocity; (Figure 3E) average speed; or (Figure 3F) resting time (Figures 3C–F). Improvements of the cognitive decline were observed with recognition object (short term memory test) in animals treated with TW1 that spent more time with the novel object compared to the old object (two-tailed *t*-test, *p* = 0.0019; Figure 4A). Significant differences were observed between treated vs. control groups with Closed Field Symmetrical Maze, respectively Day1 two-tailed *t*-test *p* = 0.0001; Day 2 two-tailed *t*-test *p* = 0.0015; Day 3 two-tailed *t*-test *p* = 0.0002; Figures 4B–D). Behavioral task results did not significantly differ in male and female mice.

### Immunohistology for PH1, CP13, GFAP and Iba-1

**Figure 5:**
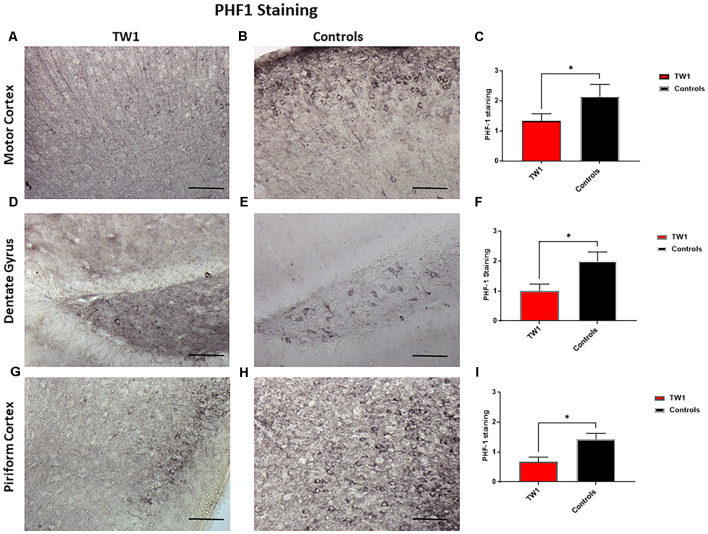
PHF1 Immunohistochemistry. TW1 passive immunotherapy reduces tau histopathology in hTau/PS1 mice model with PHF-1 PHF1 immunostaining. **(A–C)** Immunized animals had 38% less PHF-1 stained tau pathology in the motor cortex representing a trend for reduction (*p = 0.0448; one-tailed *t*-test). **(D–F)** TW1 treated mice showed a 50% reduction of PHF-1 immunoreactivity in the dentate gyrus (*p = 0.02; two-tailed *t*-test). **(G–I)** TW1 treated mice showed a 60% reduction of PHF-1 immunoreactivity in the piriform cortex (*p = 0.01; two-tailed *t*-test). Scale bar = 100 μm.

**Figure 6:**
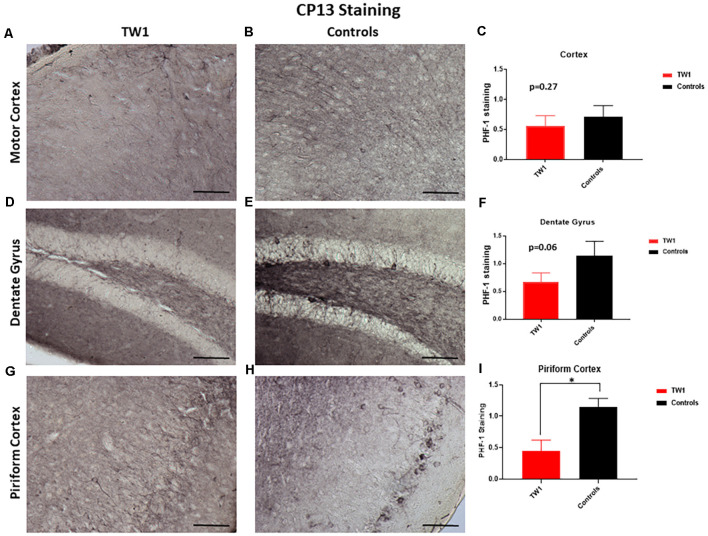
CP13 Immunohistochemistry. With CP13 immunostaining, no significant differences were observed in the motor cortex **(A–C)** and the dentate gyrus **(D–F)**. TW1 treated mice compared to vehicle controls showed reduced CP13 immunostaining in the piriform cortex (*p = 0.02; two-tailed *t*-test; **G–I**). Scale bar = 100 μm.

Brain sections were stained with two monoclonal antibodies to assess phosphorylated tau pathology: PHF1 which recognizes epitopes Ser396 and Ser404 and with monoclonal antibody CP13 that recognizes Ser202. Semi-quantitative analysis of tau pathology in the motor cortex, dentate gyrus, and piriform cortex was performed. Statistical analysis was performed with GraphPad Prism. Differences were observed between treated and non-treated animals stained with PHF-1 antibody. There is a 38% reduction of tau pathology in the motor cortex of treated mice (*p* = 0.0448; one-tailed, *t*-test; Figures 5A–C). A significant difference was seen in the dentate gyrus with a 50% reduction of PHF-1 (*p* = 0.02; two-tailed, *t*-test; Figures 5D–F) and a 60% reduction in the piriform cortex (*p* = 0.01; two-tailed, *t*-test; Figures 5G–I). CP13 immunohistochemistry detected no significant reductions in the motor cortex or the hippocampus of treated animals vs. controls (Figures 6A–F, respectively). However, a significant difference was observed in the piriform cortex of treated animals (*p* = 0.02; two-tailed *t*-test; Figures 6G–I).

**Figure 7:**
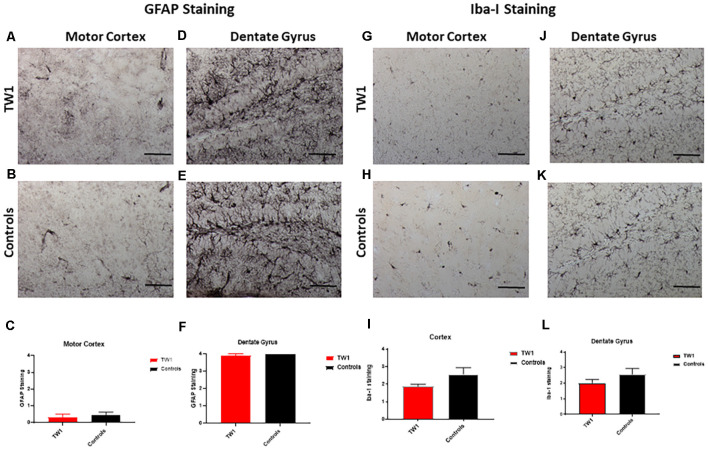
No significant differences observed with GFAP and Iba-1 Immunostaining. The semi-quantitative analysis of GFAP immunoreactivity showed no significant differences comparing TW1 and vehicle-treated controls in the motor cortex **(A–C)** or in the dentate gyrus **(D–F)**. The semi-quantitative analysis of Iba-1 immunoreactivity showed no significant differences comparing TW1 and vehicle-treated controls in the motor cortex **(G–I)** or in the dentate gyrus **(J–L)**. Scale bar = 100 μm.

Astrocytes and microglia were stained with anti-GFAP and anti-Iba-1 antibodies respectively. The semi-quantitative analysis with GFAP and Iba-1 did not show a significant difference between treated and non-treated mice (Figure 7).

There were no significant differences in the immunohistochemical results in male and female mice.

### Western Blot

**Figure 8:**
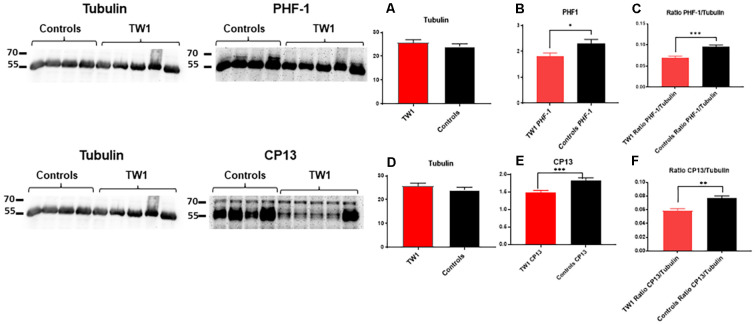
TW1 passive immunotherapy reduces biochemical tau pathology in the hTau/PS1 mice model. Densitometric analysis of the blots from the soluble fraction indicates that an equal amount of tau protein was used in treated vs. control animals **(A,D)**. PHF-1 staining shows a decrease of pathological tau in treated animals compared to the controls (*p = 0.03; two-tailed *t*-test; **B**). A significant reduction was also observed in the ratio of PHF1/Tubilin (***p = 0.0006; two-tailed *t*-test; **C**). With CP13 antibody immunoblotting a reduction of tau pathology in treated animals vs. controls is observed (***p = 0.0007; two-tailed *t*-test; **E**). A comparable effect was observed in the ratio CP13/Tubulin (**p = 0.0014; two-tailed *t*-test; **F**).

Soluble and insoluble fractions were extracted from the brains of treated and control animals. Densitometric analysis of the blots from the soluble fraction indicates an equal amount of tau protein in treated and control animals (Figure 8A). The immunoreactivity of tau was significant with PHF-1 and CP13 antibodies. PHF1 staining shows a decrease of pathological tau in treated animals compared to the controls (*p* = 0.03; two-tailed *t*-test; Figure 8B). A comparable effect was observed in the ratio of PHF1/Tubulin (*p* = 0.0006; two-tailed *t*-test; Figure 8C). Immunoreactivity with CP13 antibody in the reduction of tau pathology (test *p* = 0.0007; two-tailed *t*-test; Figure 8E). A comparable effect was observed in the ratio CP13/Tubulin (test *p* = 0.0014; two-tailed *t*-test; Figure 8F).

## Discussion

There is a paramount need to develop effective therapeutic approaches for AD, which is the only cause of death among the top ten causes of death for which there is no disease-modifying means to treat or even slow progression (Alzheimer’s Association, 2019; Long and Holtzman, 2019). The neuropathology of AD is characterized by the accumulation of amyloid β (Aβ) in the form of amyloid plaques and congophilic amyloid angiopathy, as well as the accumulation of abnormally phosphorylated tau, in the form of NFTs (Nelson et al., 2012; Long and Holtzman, 2019). Oligomeric species of both Aβ and tau are thought to be the most neurotoxic and capable of spreading pathology using prion-like mechanisms (Viola and Klein, 2015; Jucker and Walker, 2018). Numerous therapeutic avenues are being explored to treat AD; however, immunotherapeutic approaches are showing great promise (Drummond et al., 2018; Herline et al., 2018a; Kwon et al., 2020). Despite showing success in many AD models immunotherapy targeting Aβ has failed in clinical trials thus far (Reiss et al., 2020; Vaz and Silvestre, 2020). Immunotherapy addressing tau pathology has been less extensively investigated, but may represent a better target, since this pathology correlates better with AD cognitive symptoms (Nelson et al., 2012; Götz and Götz, 2019; Plotkin and Cashman, 2020; Soeda and Takashima, 2020).

In this article we show for the first time that the use of passive immunization with an anti-PrP antibody can effectively ameliorate tau-related pathology *in vivo*, using an AD model with extensive tau only pathology. Numerous studies have previously shown that Aβo mediates toxicity *via* PrP^C^ (Jarosz-Griffiths et al., 2016; Smith et al., 2019). Also, it had been shown that both the Aβo impairment of LTP *in vitro* and Aβo mediated toxicity in an amyloid model of AD could be blocked by an anti-PrP monoclonal antibody (mAb) 6D11 *in vivo* (Laurén et al., 2009; Chung et al., 2010). It also has been shown that 6D11 can inhibit PrP^Sc^ infectivity both *in vivo* and *in vitro* by blocking its interaction with PrP^C^ (Sadowski et al., 2009; Pankiewicz et al., 2019). 6D11 has the epitope PrP97-100, which is part of the epitope we document anti-PrP TW1 has, PrP 94-108 (Figure 2). This region of PrP has been demonstrated to be critical for the binding of multiple oligomeric species to PrP^C^, including Aβo and tau oligomers (Corbett et al., 2020). Hence this is likely the reason why TW1 is effective at reducing pathology in our tau-only pathology hTau/PS1 Tg AD model. Recent studies using mouse primary neurons and iPSC-derived human neurons have shown that tau oligomer binding to PrP^C^ mediates disruption of LTP and neurotoxicity (Ondrejcak et al., 2018; Corbett et al., 2020). In the current study, we are the first to show that blocking this interaction* in vivo* can be therapeutically effective for ameliorating tau-related pathology. We also document the sequence of the PrP^C^ binding paratope of TW1 (Figure 1). This information could be used to design antibody mimetics or peptoids with a similar structure and binding affinity to PrP^C^, which could also have therapeutic activity.

Importantly we show that TW1 treatment does not affect the motor function of mice using the traverse beam, rotarod, and measures of activity (Figure 3). The presence of motor dysfunction would be a sign of potential toxicity and can confound interpretation of behavioral testing. Some tau only Tg models have motor dysfunction; however, this had not been noted in our hTau/PS1 Tg AD model (Boutajangout et al., 2008; Drummond and Wisniewski, 2017; Götz and Götz, 2019). This model expresses mutated PS1 (M146L) and all human tau isoforms on a murine tau knockout background (hTau/PS1/mTau−/−). We choose to use this model as it has an early onset of tau pathology (at ~3 months) without any motor deficits (Boutajangout and Wisniewski, 2014). This model has been well characterized and previously used to study screen AD therapies (Boutajangout et al., 2008, 2009, 2010). After the relatively short 3 month period of treatment with TW1 cognitive benefits on both the spontaneous ORT and the closed field symmetrical maze. ORT is a short term memory test (Boutajangout et al., 2012). On ORT testing wild type mice normally spend more time exploring the novel object; however, the vehicle Tg mice spend equal time with the old and novel objects, while the TW1 treated mice showed a preference for the novel object (*p* = 0.0019; Figure 4A). The closed field symmetrical maze measures spatial/working memory (Boutajangout et al., 2012), with TW1 treated mice also performing significantly better compared to vehicle mice on all 3 days of testing. These findings on two cognitive tasks indicate TW1 treatment produces robust behavioral benefits in this model with extensive tau pathology.

TW1 treatment resulted in clear reductions of phosphorylated tau immunoreactivity using both CP13 and PHF-1. These two antibodies are among the most characterized identifiers of neurofibrillary tangle pathology. TW1 treated mice showed reductions in PHF1 in all areas examined which included the motor cortex, dentate gyrus, and piriform cortex, with 38%, 50%, and 60% reductions respectively (Figure 5). Significant reductions with TW1 using CP13 were limited to the piriform cortex. CP13 recognizes Ser202 on phosphorylated tau, while PHF1 recognizes Ser396 and Ser404 (Koss et al., 2016). Studies have shown that CP13 immunoreactivity is a very early phosphorylation change on tau that can be seen in otherwise normal-looking neurons, while PHF1 immunoreactivity occurs later in disease progress (Koss et al., 2016). Our immunohistochemical results suggest that TW1 treatment is more effective at blocking the later stages of tau hyperphosphorylation that are detected with PHF1, as compared to the earlier stages detected by CP13. Biochemically we confirmed that TW1 treatment results in a significant and robust reduction of PHF1 immunoreactive tau species (Figures 8A–C). This reduction was also robust on CP13 immunoreactive tau aggregates (Figures 8D–F) suggesting that TW1 is also able to inhibit earlier stages of tau hyperphosphorylation to some degree.

Since TW1 is an anti-PrP antibody one can question as to what is the mechanism by which it reduces tau pathology? In our prior studies using 6D11 in APP/PS1 mice to block Aβo toxicity by preventing the interaction with PrP^C^, we documented cognitive benefits and less synaptic loss; however, the amyloid burden was not significantly reduced (Chung et al., 2010). It is well documented that a significant mechanism of tau pathology spread is *via* prion-like processes (Jucker and Walker, 2018; Gibbons et al., 2019). We believe it is likely that this prion-like spread of tau pathology is also in part dependent on PrP^C^. Importantly we have previously shown that the emergence and extent of tau pathology following traumatic brain injury (TBI) correlates with PrP^C^ expression. Knock out of PrP^C^ completely blocks the emergence of tau pathology following TBI, while overexpression allows for the development of much greater and more wide-spread pathology (Rubenstein et al., 2017). Hence in the current experiment, we suggest that tau pathology in the TW1 treated mice was reduced by the inhibition of tau oligomer prion-like spread. This hypothesis needs to be more fully tested in models of tau prion propagation.

Tau pathology targeted immunotherapy is a less explored area of investigation compared to Aβ targeted approaches; however, it is a very active area of study with two trials of active immunization and ten on-going passive immunization trials (Götz and Götz, 2019; Plotkin and Cashman, 2020; Soeda and Takashima, 2020; Vander Zanden and Chi, 2020). An important criterion for this approach is that the immune response generated must avoid interacting with native, monomeric tau in neurons; hence, the majority of the anti-tau antibodies being tested are more specific to aggregated, oligomeric tau species (Plotkin and Cashman, 2020; Soeda and Takashima, 2020; Vander Zanden and Chi, 2020). These anti-tau antibodies aim to mainly act in the extracellular space, to stop the prion-like spread of tau oligomer pathology. Our approach of targeting the tau oligomer to PrP^C^ is similar in these respects, as it also avoids any interaction with native tau and acts in the extracellular space. However, a significant potential advantage of our approach is that it can block the interaction of multiple oligomeric species that bind to PrP^C^ and it does not specifically target just the tau oligomer interaction.

Importantly we have shown that our treatment with TW1 does not induce CNS inflammation by measuring astrocytosis by GFAP and microgliosis with Iba-1. No differences in these classical markers of brain inflammation were noted comparing TW1 treated vs. control mice (Figure 7). PrP^C^ is a self-protein expressed on the cell membrane of neurons; hence there is a clear risk of auto-immune toxicity. Certainly, it has been shown that intracranial administration of some anti-PrP monoclonal antibodies induces neuronal apoptosis (Solforosi et al., 2004; Tayebi and Hawke, 2006). However, several other studies using different anti-PrP antibodies did not show significant toxicity (Klöhn et al., 2012; Xanthopoulos et al., 2013; Wisniewski and Goñi, 2018). A study using a panel of anti-PrP antibodies it was demonstrated that toxicity may be PrP epitope dependent; with antibodies with an epitope similar to 6D11 or TW1 showing no evidence of toxicity (Sonati et al., 2013). In our studies of passive immunization with anti-PrP antibodies such as 6D11 (which has a similar epitope to TW1) and other mAbs, we have not seen evidence of any significant toxicity (Sigurdsson et al., 2002; Wisniewski et al., 2002; Pankiewicz et al., 2006; Spinner et al., 2007; Sadowski et al., 2009; Chung et al., 2010). Studies using ICSM35, an anti-PrP antibody with an epitope of PrP 95-105, has also been shown to result in cognitive benefits, without toxicity, in a rat Tg model of AD with amyloid plaques (Zhang et al., 2017). Passive and active immunization targeting PrP is emerging as a potential therapeutic approach for prion disease and current experience suggests that this can be done safely, but with certain caveats that include consideration of epitopes targeted, dosage, and routes of administration (Wisniewski and Goñi, 2018; Ma and Ma, 2020).

## Conclusion

We present the first *in vivo* demonstration that targeting PrP^C^ is a viable therapeutic strategy to address tau-related pathology. Significantly, prior work has shown that targeting PrP^C^ in this manner can also ameliorate Aβo mediated toxicity (Chung et al., 2010; Salazar and Strittmatter, 2017). In our past work, we have suggested that therapeutic approaches that concurrently target Aβ and tau pathological pathways are the ones with the highest translatability potential to effectively treat AD in patients (Wisniewski and Goñi, 2015; Wisniewski and Drummond, 2019). We are currently also developing mAbs that concurrently target Aβ and tau oligomers (Goñi et al., 2017, 2018; Herline et al., 2018b); hence there are several non-mutually exclusive means of achieving this goal. PrP^C^ also appears to be important for binding other oligomeric species including α-synuclein (Corbett et al., 2020); therefore targeting this interaction has the potential for also addressing these pathologies. The majority of AD patients at autopsy have mixed pathologies, with TDP-43 and α-synuclein related lesions being common (Schneider et al., 2007; James et al., 2016; White et al., 2016; Spires-Jones et al., 2017). Hence effective treatment of AD may require addressing each of these pathologies concurrently to achieve optimal cognitive benefits. We show that immunotherapeutic targeting of PrP^C^ to treat tau pathology is effective *in vivo*, without apparent toxicity; we believe this approach, with further development, has great promise to translate to AD patients.

## Data Availability Statement

The original contributions presented in the study are included in the article, further inquiries can be directed to the corresponding authors.

## Ethics Statement

The animal study was reviewed and approved by NYU Grossman School of Medicine’s Institutional Animal Care and Use Committee (IACUC).

## Author Contributions

AB performed experiments and helped write the article. WZ, FP, JK and WA performed experiments. TW wrote the article and supervised experiments. All authors contributed to the article and approved the submitted version.

## Conflict of Interest

AB and TW are inventors on a NYU Grossman School of Medicine issued USPTO patent (10,662,246) covering the sequence and uses of TW1. The remaining authors declare that the research was conducted in the absence of any commercial or financial relationships that could be construed as a potential conflict of interest.
